# Valbenazine for the Treatment of Chronic Motor or Vocal Tic Disorder (CMVTD)

**DOI:** 10.7759/cureus.92968

**Published:** 2025-09-22

**Authors:** Adam Bied, Anant Akash Sakthivel, Ritvij Satodiya

**Affiliations:** 1 Psychiatry, HKD Outreach, Mattapan, USA; 2 Psychiatry, Sri Ramachandra Institute of Higher Education and Research, Chennai, IND; 3 Psychiatry, New York University Grossman School of Medicine, New York, USA

**Keywords:** chronic motor vocal tic disorder, psychopharmacology, tic disorder and psychiatry, tic disorders, valbenazine

## Abstract

Chronic motor or vocal tic disorder (CMVTD) is a distressing neuropsychiatric condition. A subset of patients remains refractory to currently approved therapies. The present report describes a pediatric patient with CMVTD who had previously failed multiple pharmacological treatments, including two Food and Drug Administration (FDA)-approved agents (pimozide, aripiprazole), guanfacine, psychotherapy, and novel compounds (lumateperone, cariprazine). The patient was initiated on valbenazine, titrated to 80 mg daily. Clinical improvement was noted within 24-48 hours, with sustained benefit and good tolerability. Tic severity, measured by the Yale Global Tic Severity Scale-Revised (YGTSS-R), improved from 65 at baseline to five following treatment (net reduction of 60 points). This case illustrates a marked therapeutic response to valbenazine in a patient with CMVTD refractory to conventional and novel therapies. These observations highlight the potential role of vesicular monoamine transporter 2 (VMAT2) inhibition in tic disorders and also the need for further clinical study.

## Introduction

Chronic motor or vocal tic disorder (CMVTD) is characterized by motor or vocal tics persisting for more than one year, with its onset before the age of 18 years [1]. Three medications are approved by the Food and Drug Administration (FDA) for tic disorders: pimozide, haloperidol, and aripiprazole [2]. An additional 11 medications are endorsed by the American Academy of Child and Adolescent Psychiatry (AACAP). These include seven other neuroleptics, benzodiazepine, clonazepam, the two formulations of clonidine and guanfacine, and the vesicular monoamine transporter (VMAT) tetrabenazine [3]. Of these, two - tiapride and sulpiride - are not commercially available in the authors’ home nation, the United States, and a third, ziprasidone, is only commercially available in doses above those recommended by the AACAP. Some patients are either intolerant of, or insufficiently responsive to, these medications [4]. In circumstances in which this happens, it is unclear which approach to management is appropriate.

Valbenazine is a novel medication, which was granted Breakthrough Drug Status by the FDA in 2015, received its first FDA approval for tardive dyskinesia two years later, and received its second FDA approval, for Huntington’s Disease in 2023 [5-7]. Valbenazine is a prodrug, comprised of an ester of [+]-α-dihydrotetrabenazine (DTBZ) with the amino acid L-valine [8]. It is extensively hydrolyzed to the active metabolite DTBZ and displays a half-life of 15 to 22 hours [8]. Theorized to induce reductions in dopamine release by the inhibition of pre-synaptic VMAT2, valbenazine has been shown to have little affinity for other transporters or receptors [9]. Consistent with this, reduced VMAT2 activity appears to decrease the loading of dopamine into synaptic vesicles, reducing the downstream release of that neurotransmitter, and ultimately decreasing the concentrations of dopamine in the synaptic cleft [10,11]. An unsuccessful phase 2B clinical trial of this medication for Tourette’s syndrome (TS) was completed in 2018 [12]. While the medication remains under investigation for multiple other indications, there are no ongoing or published trials of valbenazine for any other tic disorder diagnoses [13]. A meta-analysis published in 2022 assessed VMAT2 inhibitors as a treatment of various kinds of tics [14]. This article offers a tacit endorsement of the strategy but also lacks data specific to CMVTD [14]. The authors hypothesize that valbenazine may be a particularly effective choice for CMVTD, and present here a case describing this treatment strategy.

## Case presentation

The patient is an 11-year-old boy who presented to the lead author’s clinic (AB) for treatment of involuntary muscle contractions in the face and neck. Described as present for at least several years, the patient had neither been assessed nor treated for this complaint prior to presentation at the clinic. After obtaining history, the diagnosis of CMVTD was derived with the presence of pronounced motor tics concentrated in the face and to a lesser extent, the neck. The patient underwent a series of medication treatments as well as psychotherapy over the ensuing two years. Treatments included pimozide, guanfacine, aripiprazole, and cariprazine with generally poor treatment response (see Table 1). 

**Table 1:** Medication Management for Chronic Motor or Vocal Tic Disorder (CMVTD)

Measures	Timeline (Days)																																
Time	~Multiple Years	-880 days	-875 days	-867 days	-852 days	-751 days	-737 days	-721 days	-707 days	-690 days	-650 days	-608 days	-401 days	-386 days	-366 days	-338 days	-309 days	-287 days	-217 days	-188 days	-71 days	-41 days	0 days	+2 days	+8 days	+11 days	+14 days	+23 days	+27 days	+28 days	+42 days	+44 days	+46 days
Clinical Intervention	Pretreatment	Pimozide 0.5 mg daily	Pimozide 1 mg daily	Pimozide 2 mg daily	Psychotherapy initiated	Reduced pimozide to 1 mg daily, guanfacine 1 mg daily initiated	Guanfacine increased to 1 mg BID, Maintained pimozide 1 mg daily	Guanfacine increased to 2 mg qAM / 1 mg qHS. Maintained pimozide 1 mg daily	Guanfacine suspended. Maintained pimozide 1 mg daily	Pimozide 1 mg suspended	Resumed pimozide 1 mg daily	Increased pimozide to 2 mg daily	Suspended pimozide 2 mg daily, Initiate lumateperone 42 mg daily	Lumateperone 42 mg daily discontinued	Initiate aripiprazole 5 mg daily	Increased aripiprazole to 10 mg daily	Increase aripiprazole to 15 mg daily	Increase aripiprazole to 20 mg daily	Increase aripiprazole to 30 mg daily	Increase aripiprazole to 40 mg daily	Aripiprazole was reduced to 20 mg daily and cariprazine 1.5 mg daily was initiated	Aripiprazole 20 mg daily was discontinued and caripiprazine raised to 3 mg daily	Initiate valbenazine 40 mg daily. Continue cariprazine 3 mg daily.	No treatment changes	No treatment changes	No treatment changes	Increase valbenazine to 60 mg daily. Continue cariprazine 3 mg daily.	No treatment changes	No treatment changes	Increase valbenazine to 80 mg daily. Continue cariprazine 3 mg daily	Continue valbenazine 80 mg daily. Reduce cariprazine to 1.5 mg daily	Continue valbenazine 80 mg daily. Suspend cariprazine 1.5 mg daily	Continue valbenazine 80 mg daily
Clinical Manifestations	Motor Tics	Subjective improvement in tics	Subjective improvement in tics	Subjective improvement in tics, sedation	No obvious clinical change	Improvement in sedation, worsening tics	Modest subjective improvement in tics	Modest subjective improvement in tics	Patient experienced syncope on guanfacine	Patient experienced more pronounced tics, less sedation.	Tics improve subjectively	Sedation with minimal improvement in tics	Minimal improvement in tics, syncope manifested	Tics worsened, syncope resolved with discontinuation of lumateperone	No change in tics though no obvious side effects to aripiprazole	No obvious changes in tics.	Minimal subjective improvement in tics	Minimal subjective improvement in tics	Minimal to no subjective improvement in tics.	No subjective improvement in tics	No obvious improvement in tics	No obvious improvement in tics	Poor treatment response trigger medication changes	Subjective reduction in tics	Subjective reduction in tics	Subjective reduction in tics	Subjective reduction in tics	Subjective reduction in tics	Subjective reduction in tics	Subjective reduction in tics	Subjective reduction in tics	Subjective reduction in tics	Subjective reduction in tics. Improved wakefulness
Yale Global Tic Severity Scale-Revised (YGTSS-R): Total Score	-	-	-	-	-	-	-	-	-	-	-	-	-	-	-	-	-	-	-	-	-	-	65	32	32	22	30	22	15	-	15	-	5

Following diagnosis, the patient was initiated on pimozide (0.5 mg daily). Over subsequent visits, this medication was titrated to a maximum dose of 2 mg daily. Due to poor treatment response, immediate-release guanfacine was subsequently added. The patient continued to display pronounced tics on this drug combination and subsequently manifested presyncope, leading to the cessation of the guanfacine. Alternative treatment options were reviewed and the novel neuroleptic, lumateperone (42 mg daily), was subsequently initiated. After three days on the medication, during which tics persisted and the patient re-experienced symptoms of presyncope, the drug was suspended. Aripiprazole was subsequently employed. Over multiple visits, the drug was incrementally increased from a starting dose of 5 mg, to a final dose of 40 mg, daily. The tics persisted throughout this trial and alternative treatments were again examined. A cross-titration off of aripiprazole and onto cariprazine followed with the latter titrated to a maximum dose of 3 mg daily.

A Yale Global Tic Severity Scale Revised (YGTSS-R) was subsequently employed, revealing a score of 65 (0 day/Med/Dose: cariprazine 3 mg daily [15]. Note: For illustrative purposes, the time frame was set to day zero by the authors). Valbenazine 40 mg daily was then initiated. Serial YGTSS-R were done, revealing scores of 32 (+2 days), 32 (+8 days) and 22 (+11 days). Valbenazine was increased to 60 mg daily (+14 days) and serial YGTSS-R followed with scores of 30 (+14 days), 22 (+23) and 15 (+27 days). Valbenazine was increased further to 80 mg daily (+28 days). A follow-up YGTSS-R done later revealed a score of 15 (+42 days). Cariprazine, dosed at 3 mg daily, was employed throughout this period of treatment but was reduced to 1.5 mg daily and later (+44 days) discontinued. A subsequent YGTSS-R revealed a score of 5 (+46 days). No adverse effects were described during administration of valbenazine, including specifically reviewed sleep, gastrointestinal, and mood symptoms.

## Discussion

This is the first published case describing a successful treatment of CMVTD with valbenazine. This report adds useful information to the currently limited literature on tic disorders and provides a preliminary clinical insight into the potential role of vesicular monoamine transporter 2 (VMAT2) inhibitors in CMVTD management. In this case, symptom improvement was observed within 24-48 hours of initiation, consistent with valbenazine’s pharmacokinetics (half-life 15-22 hours) [6]. The response was robust (YGTSS-R reduction of 60 points, pre-treatment: 65, post-treatment: 5) and the medication was generally well tolerated. Notably, benefit was observed despite prior no response to the two FDA-approved drugs for the condition (pimozide, aripiprazole), a non-FDA-approved, AACAP-endorsed treatment (guanfacine) [3], psychotherapy, and two novel agents (lumateperone, cariprazine).

Central to this case are the distinctions between TS and CMVTD. The Diagnostic and Statistical Manual of Mental Disorders, Fifth Edition, Text Revision (DSM-5-TR) identifies CMVTD and TS as separate diagnoses, with the former manifesting either motor or vocal tics, but not both, and the latter manifesting both motor and vocal tics [1]. In the phase 2B trial of valbenazine for TS, the treatment was found to outperform that of the placebo arm, but to a degree that was “lower than anticipated” [16]. In our report, a robust improvement (YGTSS-R: -60 (pre-treatment: 65, post-treatment: 5)) was observed. Two possible hypotheses for this observation may be proposed. The first is that consistent with DSM-5-TR, the distinction between TS and CMVTD shows that they are not only distinct diagnoses but different disorders. It is possible that this may explain why the treatment conceivably worked. This case raises the possibility that the diagnostic differences between TS and CMVTD may contribute to treatment variability. However, mechanistic explanations remain speculative and require further studies.

Our observation raises the question of whether valbenazine may have differential effects on motor versus vocal tics. Although current trials and meta-analyses typically report only aggregate YGTSS data [14], reporting motor and vocal sub-scores separately could help clarify potential subtype-specific responses. Another analogous approach may be to conduct trials comprising participant arms with TS, CMVTD experiencing vocal tics only, and CMVTD experiencing motor tics only, with the respective data reported for each cohort. This may then clarify whether differential treatment responses exist.

Another subject of relevance to this review is the distinction between valbenazine and its alternatives. At present, there are no FDA-approved VMAT2 inhibitors for tic disorders and only tetrabenazine has been endorsed by the AACAP for this purpose. Tetrabenazine displays a shorter half-life (3-8 hours vs. 18-22 hours) for the active metabolite and displays heterogeneity in its metabolism [17,18]. AACAP, in its practice parameters, references the agent only briefly, advising clinicians of the significant potential for ‘sedation, weight gain, depression’ as well as recommending ‘CYP2D6 pharmacogenomic testing’, recommendations not shared by the FDA for valbenazine [3]. It can also be noted that AACAP has not introduced updated practice parameters since December 2013, four years prior to valbenazine’s initial FDA approval. These differences suggest that valbenazine may represent a practical alternative in cases where tetrabenazine is not tolerated or feasible, though controlled studies are needed to establish comparative efficacy. As this is a single case, the findings should be interpreted cautiously, and replication in further studies will be necessary.

## Conclusions

This case demonstrated the robust and well-tolerated response to valbenazine in an individual with CMVTF who had failed multiple prior therapies. Though encouraging, the findings are limited by the single-case design. Larger studies are needed to establish the safety and efficacy of valbenazine in tic disorders, including potential differences in response between motor and vocal tics as well as the potential role of VMAT2 inhibition.
